# Supplementary material for: Decoding coral resistance to eutrophication through the association of hyper‑efficient denitrifiers as key microbial allies
Source: Nat Commun. 2026 May 19;17:3938. doi: 10.1038/s41467-026-72571-w (PMC13187470; doi:10.1038/s41467-026-72571-w)
Supplement: Supplementary file 1 — Supplementary Information [file 41467_2026_72571_MOESM1_ESM.pdf]

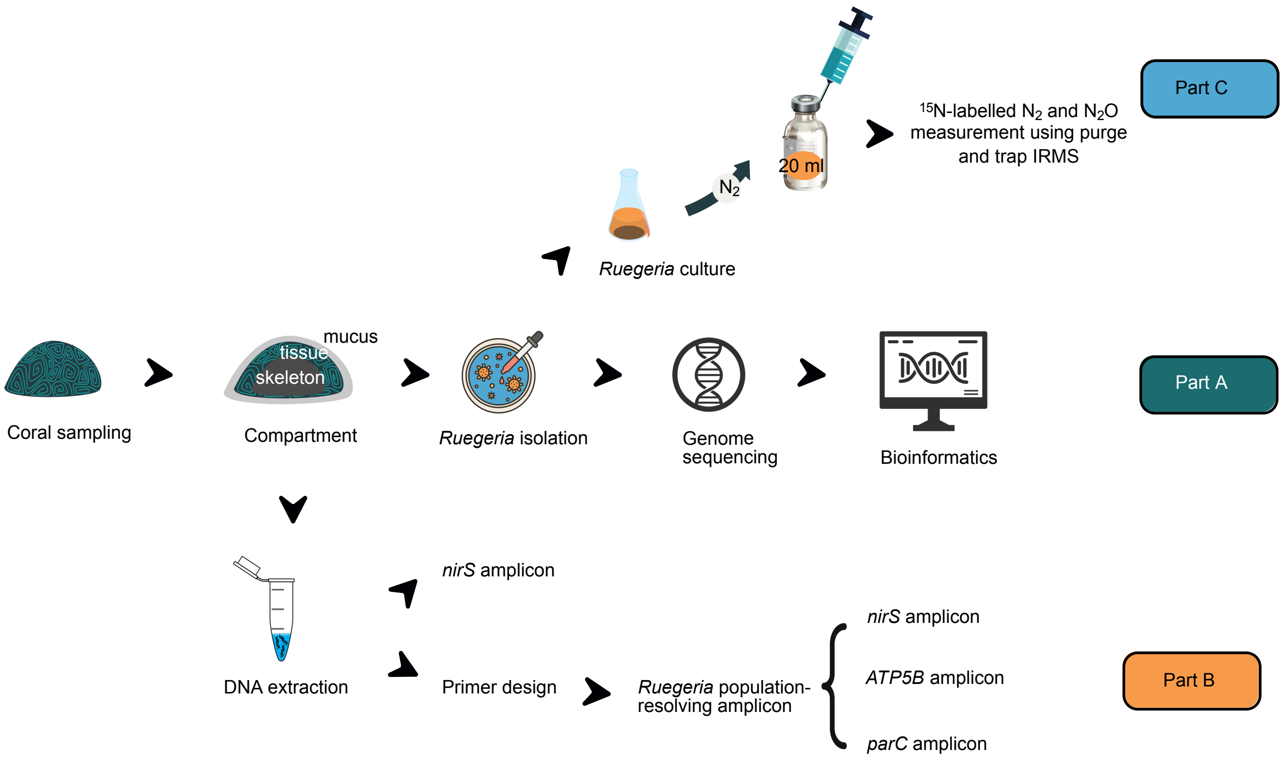

Supplementary Figure S1. Graphic summary for the experimental design. Schematic overview of the multidisciplinary research components, including culture-dependent *Ruegeria* whole-genome sequencing, culture-independent *Ruegeria* population-resolving gene amplicons, and denitrification activity measurements using the ^{15}N-stable isotope assay.

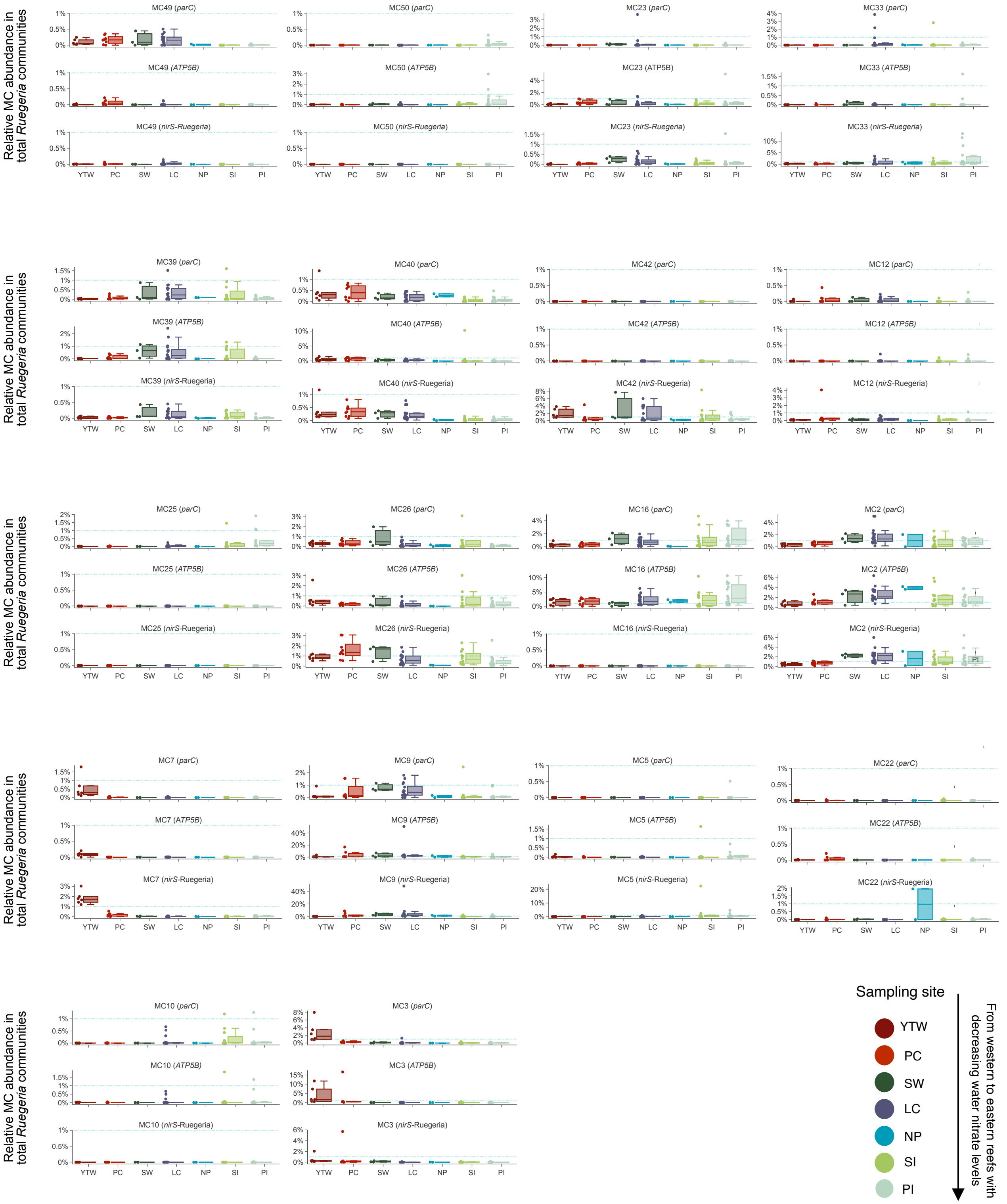

Supplementary Figure S2. **Relative abundances of different Ruegeria populations (i.e., main clusters, MC) in corals across coastal Hong Kong reefs.** One dashed line denoted the threshold of 1% relative MC abundance in total *Ruegeria* communities. Box plots were used to display maximum, median, and minimum data with scatterplots depicting the distribution of raw data (n = 3-12 for each group). Abbreviations for sampling sites were the same as those in Figure 1.

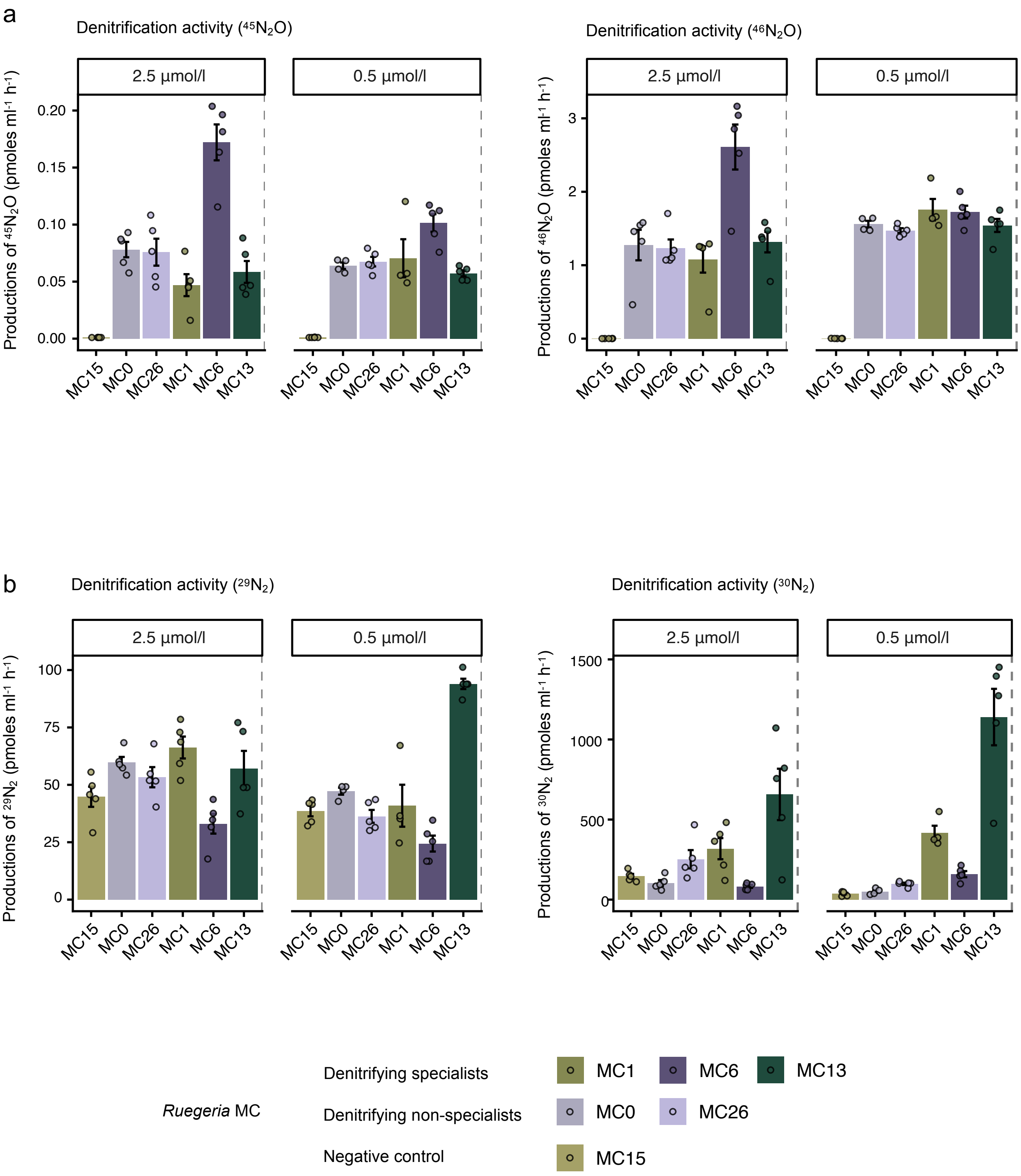

Supplementary Figure S3. **Experimental validation of the ^{15}N-stable isotope assay for quantifying denitrification activities in *Ruegeria* isolates under suboxic (2.5 µmol l^{-1} DO) and nanoxic (0.5 µmol l^{-1} DO) conditions.** Activities were assayed in three representative denitrifying specialists (MC1, MC6, MC13), two representative denitrifying non-specialists (MC0, MC26), and MC15 lacking denitrification genes as a negative control. (**a**) Production rates of $^{45}N_2O$ and $^{46}N_2O$. (**b**) Production rates of $^{29}N_2$ and $^{30}N_2$. Data were shown with the mean values and standard errors (n = 5 biological replicates each), with scatterplots depicting the distribution of raw data (n = 5 for each group). Each pair of groups was compared using a two-tailed *P* value (n = 5 for each test).

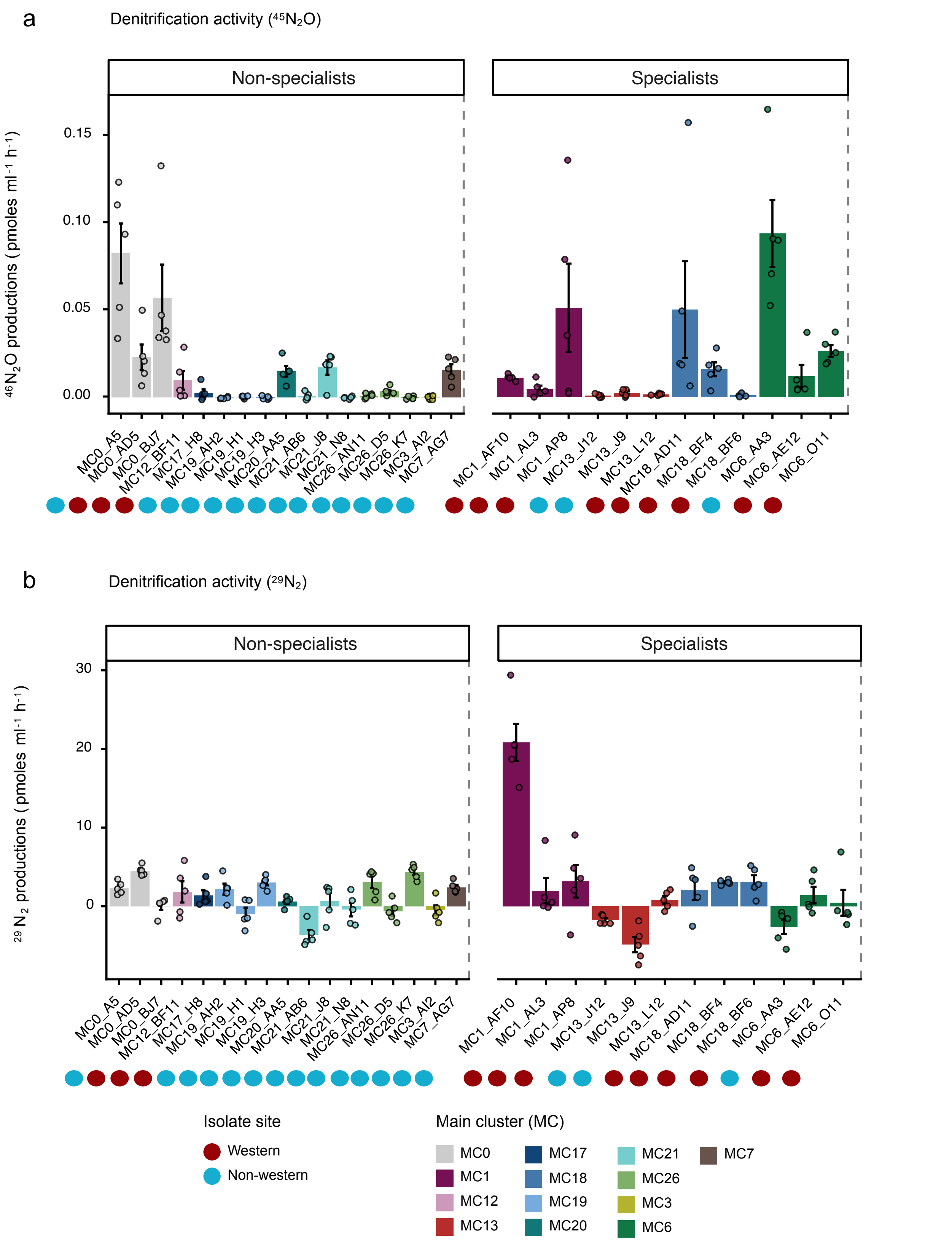

Supplementary Figure S4. Denitrification activity of *Ruegeria* populations under the nanoxic (0.5 μmol l^{-1} dissolved oxygen) condition. (**a**) $^{45}N_2O$ and (**b**) $^{29}N_2$ production rates in denitrifying *Ruegeria* specialists (four MCs) and non-specialists (nine MCs) under the ecologically relevant nanoxic (0.5 μmol l^{-1} dissolved oxygen) condition. Data were shown with the mean values and standard errors (n = 5 biological replicates each) with Y-axis scale in $^{29}N_2$ productions two orders of magnitude higher than in $^{45}N_2O$ productions. Coral sampling sites for obtaining *Ruegeria* isolates were indicated in solid blue circles for non-western sites (i.e., SW, LC, NP, and BI) and solid red circles for western site (i.e., YTW). Data were shown with the mean values and standard errors (n = 5 biological replicates each), with scatterplots depicting the distribution of raw data (n = 5 for each group). Each pair of groups was compared using a two-tailed *P* value (n = 5 for each test).

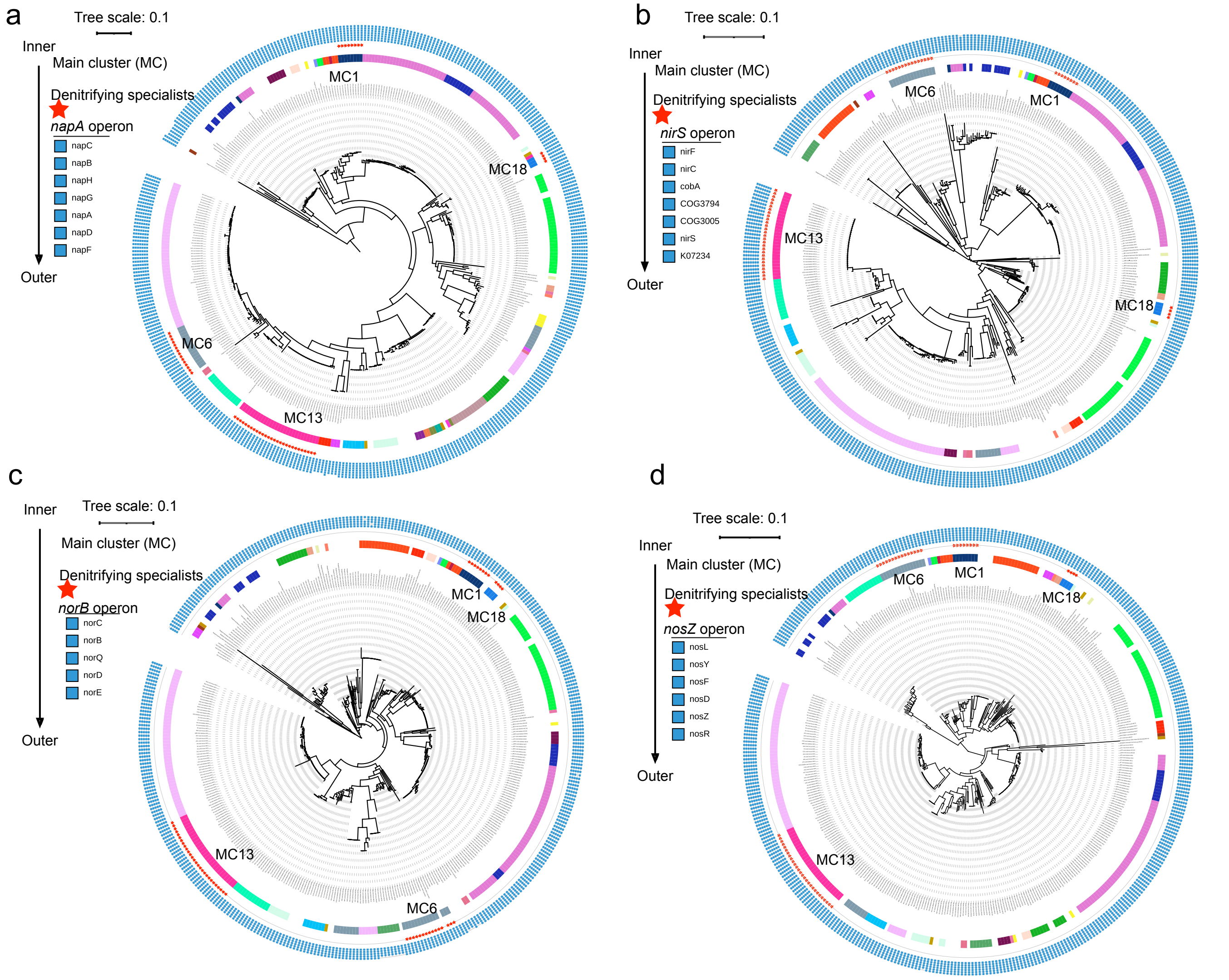

Supplementary Figure S5. Graphic summary for the experimental design. Schematic overview of the multidisciplinary research components, including culture-dependent *Ruegeria* whole-genome sequencing, culture-independent *Ruegeria* population-resolving gene amplicons, and denitrification activity measurements using the ^{15}N-stable isotope assay.

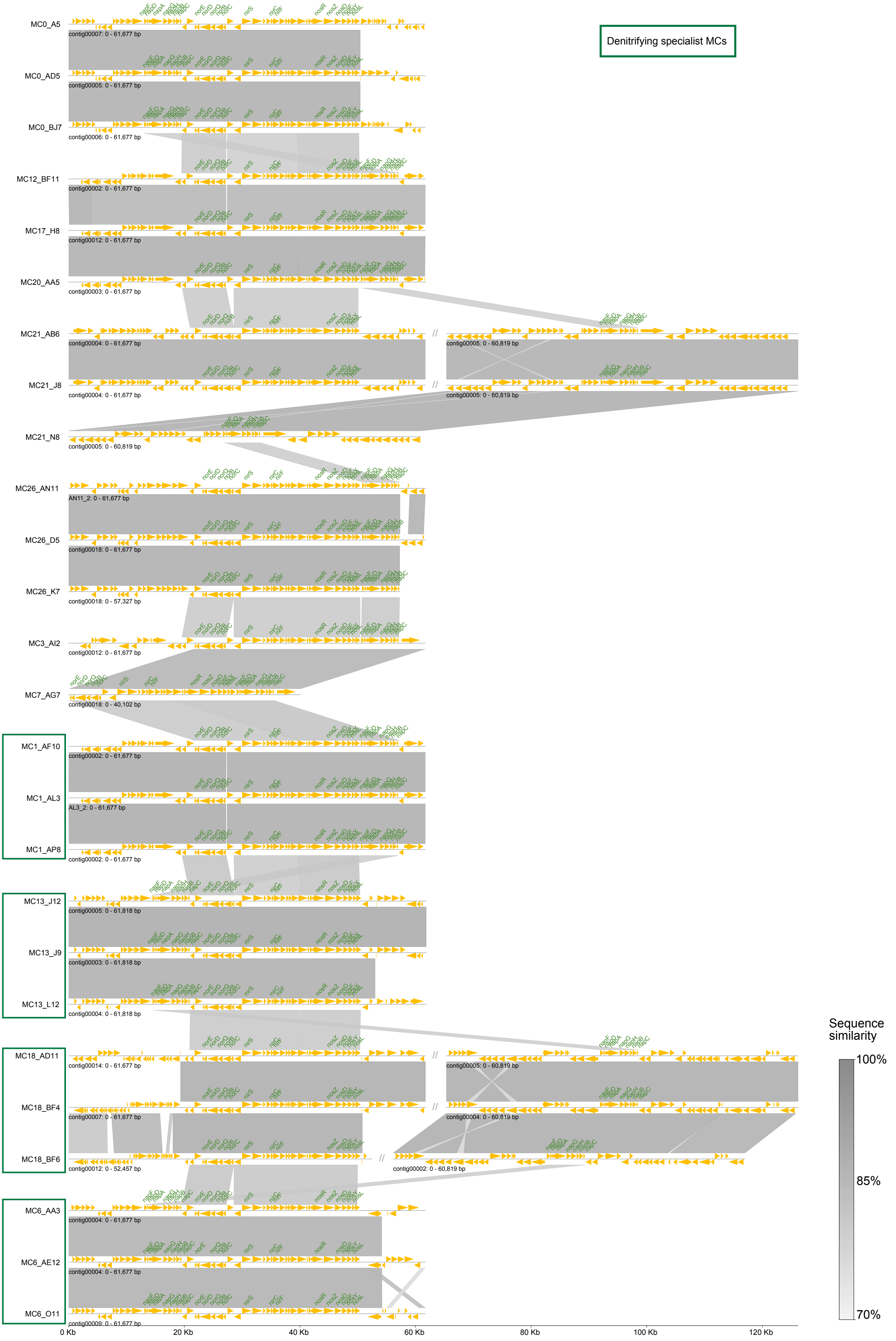

Supplementary Figure S6. Denitrification gene arrangements in *Ruegeria* genomes used in the ^{15}N-stable isotope assay. Denitrification operons were shown: *napA* (nitrate reductase), *nirS* (nitrite reductase), *norB* (nitric oxide reductase), and *nosZ* (nitrous oxide reductase). The denitrification genes were indicated in solid yellow arrows and green texts. White-grey bar displayed the sequence similarity between genomes based on BLASTn. Data sources: Next-generation sequencing.

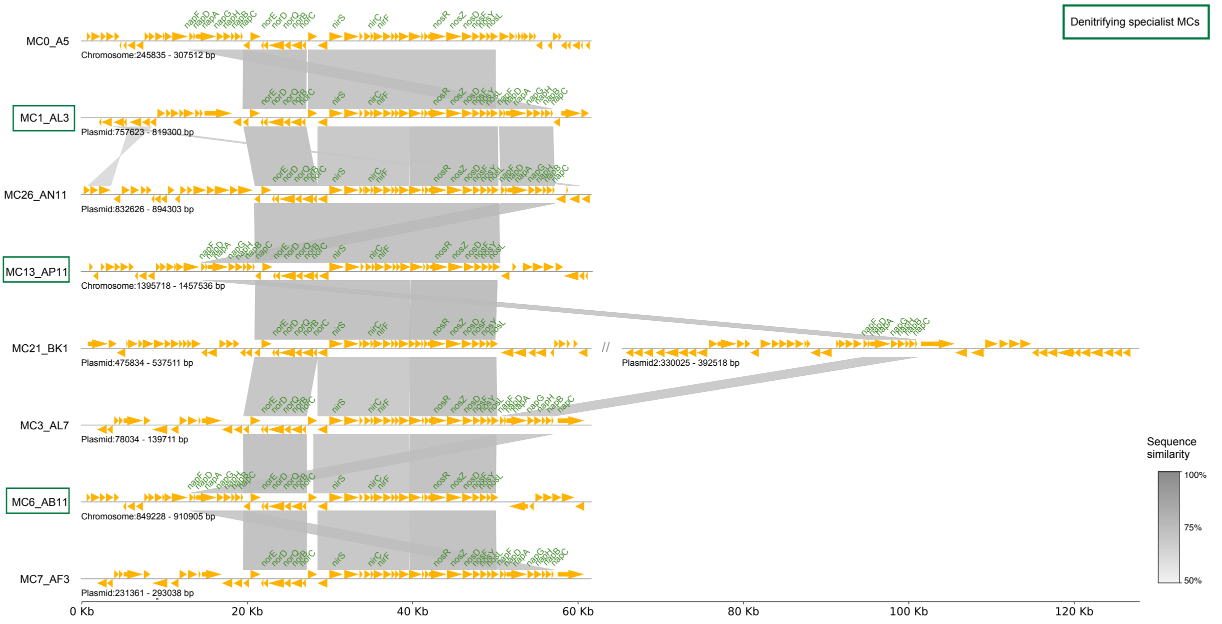

Supplementary Figure S7. Denitrification gene arrangements in eight closed *Ruegeria* genomes (affiliated to eight MCs) using in the ^{15}N-stable isotope assay. Denitrification operons were shown: *napA* (nitrate reductase), *nirS* (nitrite reductase), *norB* (nitric oxide reductase), and *nosZ* (nitrous oxide reductase). The denitrification genes were indicated in solid yellow arrows and green texts. White-grey bar displayed the sequence similarity between genomes based on BLASTn. Chromosome and plasmid were shown. Data sources: Nanopore sequencing.

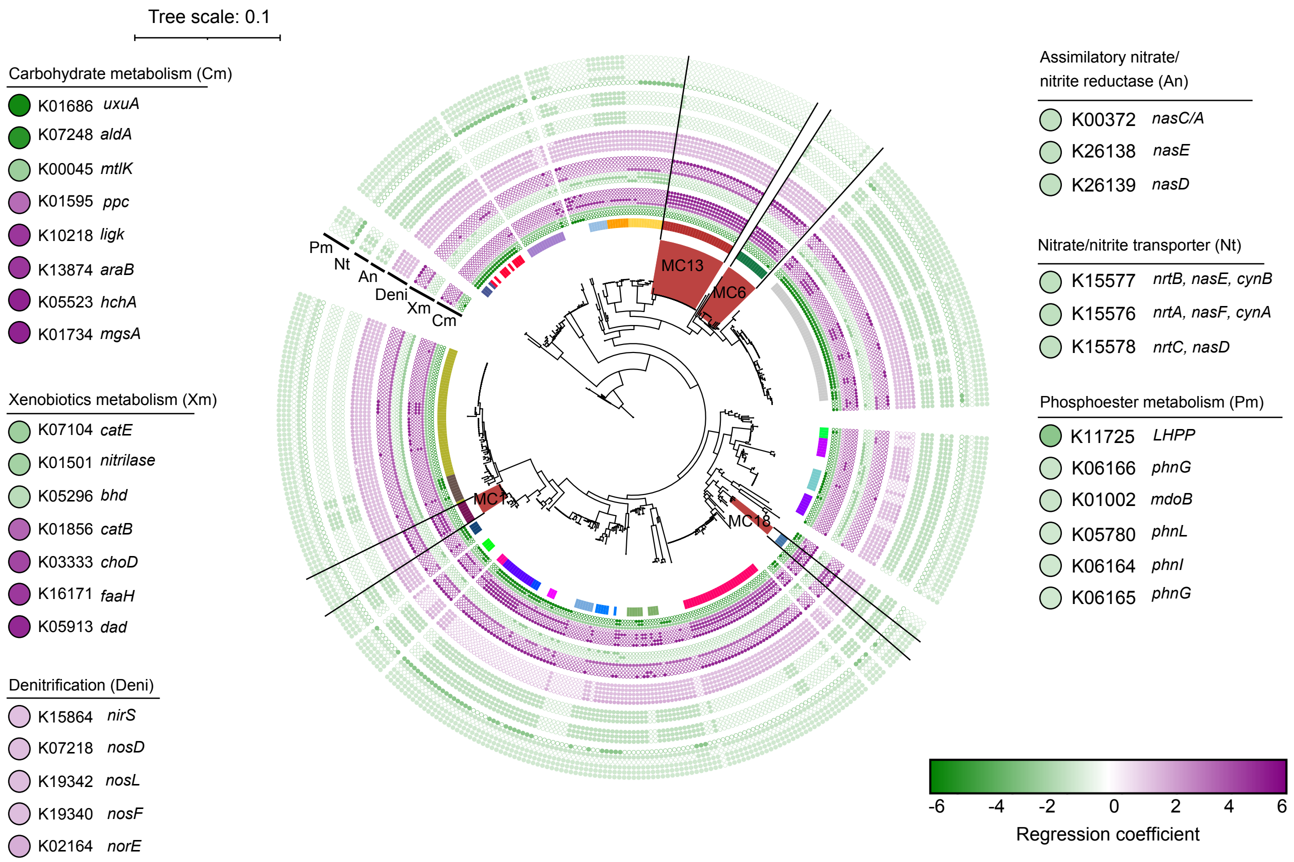

Supplementary Figure S8. Phylogenomic tree of 419 *Ruegeria* isolates showing orthologous genes (OGs) that differed in presence and absence patterns between denitrifying specialists and non-specialists. The phylogenomic tree was constructed with IQ-TREE based on concatenated single-copy orthologous genes at the amino acid level. The gene association analysis was conducted with phylogenetic signal estimation (Phylosig) and phylogenetically informed modeling (BinaryPGLMM). Pathways (i.e., carbohydrate and xenobiotics metabolisms) involved in significant gene replacements between denitrifying specialists and non-specialists were shown. Nitrogen (i.e., denitrification, assimilatory nitrate/nitrite reductase, nitrate/nitrite transporter) and phosphorus (i.e., phosphoester metabolism) cycling-related pathways involved in marginally different presence and absence patterns between specialists and non-specialist were shown. Solid and hollow circles denoted the presence and absence of each KEGG Orthology (KO) in Ruegeria genomes. Gradient purple (scale value: 0 to 6) and green colors (scale value: -6 to 0) indicated the regression coefficient from BinaryPGLMM analysis. Positive and negative values indicated the KO enrichment and depletion patterns in denitrifying specialists compared to their non-specialist counterparts, respectively. Four denitrifying specialist MCs (MC1, 6, 13, 18) were highlighted with the red background in the tree. All reported *P*-values are two-tailed.

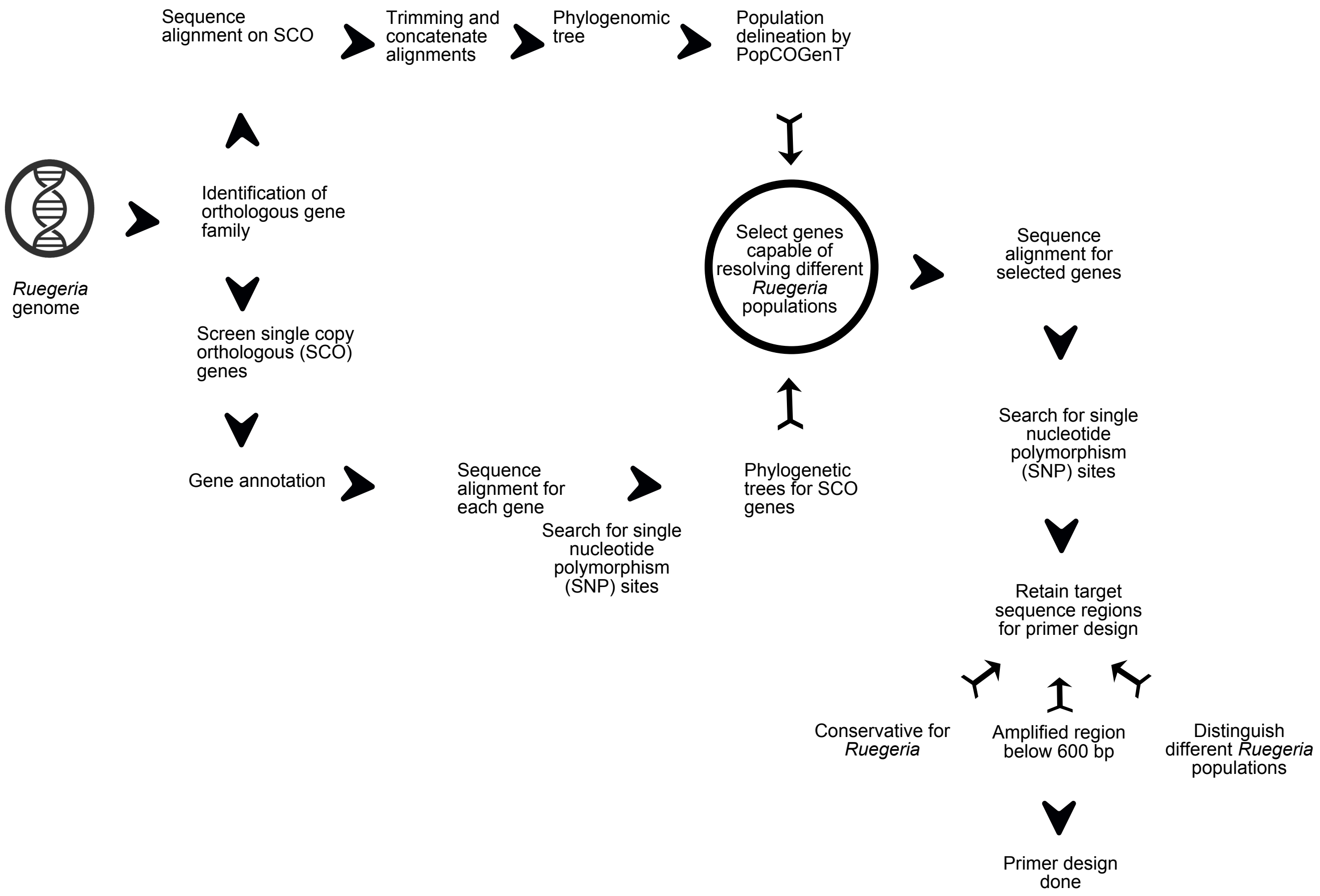

Supplementary Figure S9. Workflow showing primer design for *Ruegeria* population-resolving gene amplicons. The schematic outlines the multi-step bioinformatics pipeline used to search genes and associated amplified regions capable of distinguishing between distinct *Ruegeria* populations.
