# Supplementary material for: Decoding coral resistance to eutrophication through the association of hyper‑efficient denitrifiers as key microbial allies
Source: Nat Commun. 2026 May 19;17:3938. doi: 10.1038/s41467-026-72571-w (PMC13187470; doi:10.1038/s41467-026-72571-w)
Supplement: Supplementary file 2 — Description of Additional Supplementary Files [file 41467_2026_72571_MOESM2_ESM.pdf]

Description of Additional Supplementary Files

File name: Supplementary Data 1
Description: Water quality of seven sampling sites along coastal Hong Kong reefs. The data were shown as calculated mean values for each parameter over the course of 10 years (year 2012 to 2022).

File name: Supplementary Data 2
Description: *Ruegeria* isolate availability for the assigned populations (i.e., main clusters, MCs). Numbers of the available *Ruegeria* isolates were calculated for coral samples. Only 29 MCs (owning ≥ 3 strains per MC) were chosen for the ASV assignment in *Ruegeria* population-resolving *ATP5B*, *parC*, and *nirS* amplicons.

File name: Supplementary Data 3
Description: Metadata for 419 *Ruegeria* isolates: environmental origins (sampling site, coral species, compartment), population affiliation (i.e., main cluster, MC), genome size, completeness, contamination, and denitrification gene presence.

File name: Supplementary Data 4
Description: Information for 26 *Ruegeria* reference genomes, including species name, NCBI accession, IMG accession, and genome size.

File name: Supplementary Data 5
Description: Correlation of denitrifying specialist *Ruegeria* MCs with high-nitrate western reef sites (i.e., YTW and PC). Envfit-fitted vectors on NMDS ordination showed ^{15}N-stable isotope assayed specialist MCs (n = 4) and non-specialist MC (n = 9) directions. Cosine distances reflected vector dissimilarity to sites. R^2 indicated model fit (1 = full variance explained). The *P*-value below 0.05 indicated the statistical significance of the fitted regression models using the permutation test. The significance of envfit correlations was examined using a permutation test (empirical *P* values based on R^2) with a non-directional test of fit. One-sided/two-sided terminology is not applicable. Abbreviation for NA: MC is not available for Envfit analysis.

File name: Supplementary Data 6
Description: Correlation of water quality with *Ruegeria* population compositions in Hong Kong reef sites. Envfit-fitted vectors on NMDS ordination showed water quality parameters. Cosine distances reflected vector dissimilarity to sites. R^2 indicated model fit (1 = full variance explained). The *P*-value below 0.05 indicated the statistical significance of the fitted regression models using the permutation test. The significance of envfit correlations was examined using a permutation test (empirical *P* values based on R^2) with a non-directional test of fit. One-sided/two-sided terminology is not applicable. Abbreviation for NA: MC is not available for Envfit analysis. The water quality data were shown as calculated mean values for each

parameter over the course of 10 years (year 2012 to 2022).

File name: Supplementary Data 7
Description: Metadata for 29 *Ruegeria* isolates used in the ^{15}N-stable isotope assay. Environmental origins (site, coral species, compartment, mother colony), population affiliation, and denitrification trait.

File name: Supplementary Data 8
Description: PhylANOVA and Brownian motion evolution model analysis of ^{15}N-labelled $^{46}N_2O$ and $^{30}N_2$ productions between denitrifying specialist and non-specialist MCs. Significant differences ($P < 0.05$, *) were indicated in bold and marked with asterisks. Each pair of groups was compared using a two-tailed *P* value (n = 12 MCs for specialists, n = 17 for non-specialists).

File name: Supplementary Data 9
Description: Statistical analysis on ^{15}N-labelled $^{46}N_2O$ and $^{30}N_2$ productions by Ruegeria isolates. Linear mixed models with two fixed effects (MC and strain identities) and one random effect (replicate ID) were applied. Significance of the fixed effects was evaluated via two-way ANOVA with different lowercase letters indicating significant differences between individual isolates (two-way ANOVA with Tukey's HSD; $P < 0.05$). Each pair of groups was compared using a two-tailed *P* value (n = 5 for each test).

File name: Supplementary Data 10
Description: KEGG orthologous group functions of 2272 enriched or depleted genes in denitrifying specialist MCs. Identified via Phylosig and BinaryPGLMM analysis (threshold: n = 4 specialist MCs). Column descriptions were: Phylosig (Pagel's λ test *P*-value, ≤0.05 indicating a significant phylogenetic signal); BinaryPGLMM (Phylogenetic logistic regression *P*-value, ≤0.05 indicating a significantly specialized association); Regression coefficients (indicating binaryPGLMM effect size with positive and negative values showing enriched and depleted in specialists, respectively); Chisq test (Chi-squared test *P*-value, ≤0.05 indicating strong association without phylogeny when phylosig >0.05); Lm regression (linear regression coefficient strength with Chisq test); Significant (statistical difference with y for significant, n for non-significant, and na for failed analysis).

File name: Supplementary Data 11
Description: KEGG orthologous group functions of 80 significantly enriched or depleted genes in denitrifying specialist MCs. Identified via Phylosig and BinaryPGLMM analysis (threshold: n = 4 specialist MCs). All reported *P*-values are two-sided.

File name: Supplementary Data 12
Description: KEGG orthologous group functions of nitrogen and phosphorus cycling

genes enriched or depleted in denitrifying specialist MCs. Identified via Phylosig and BinaryPGLMM analysis (threshold: n = 4 specialist MCs). All reported *P*-values are two-sided.

File name: Supplementary Data 13
Description: KEGG orthologous group functions of 92 evolutionarily conserved genes in denitrifying specialist MCs. Identified via gene tree-species tree reconciliation using ecceTERA and ANGST (threshold: $n \geq 2$ specialist MCs).

File name: Supplementary Data 14
Description: Phylosig and BinaryPGLMM analysis on 92 evolutionarily conserved genes in denitrifying specialist MCs (retained 46 genes after filtering KOs identical between specialists vs. non-specialists). All reported *P*-values are two-sided.

File name: Supplementary Data 15
Description: Medium used for *Ruegeria* isolation from corals. Chemicals in Group A (basal medium) and B (sea salt solution) were mixed and autoclaved at 121 °C for 20 min. Chemicals in Group C (FeNaEDTA stock), D (vitamin supplement), E (carbon and nitrogen sources solution; E1, E2, or E3) were sterilized by the filtration through 0.22 µm syringe filters. Group E consisted of E1, E2, and E3 that represented three different carbon and nitrogen sources.

File name: Supplementary Data 16
Description: Minimum nutrient medium used for ^{15}N-stable isotope assay quantifying *Ruegeria* denitrification activity. Chemicals in Group A are basal salts. Chemicals in Group B are trace minerals according to the recipe of trace element solution SL-10. Chemicals in Group C are vitamins according to the recipe of the Medium No. 197.
