# Supplementary material for: Decoding coral resistance to eutrophication through the association of hyper‑efficient denitrifiers as key microbial allies
Source: Nat Commun. 2026 May 19;17:3938. doi: 10.1038/s41467-026-72571-w (PMC13187470; doi:10.1038/s41467-026-72571-w)
Supplement: Supplementary file 19 — Reporting Summary [file 41467_2026_72571_MOESM19_ESM.pdf]

nature portfolio

Corresponding author(s): Haiwei Luo

Last updated by author(s): Feb 20, 2026

Reporting Summary

Nature Portfolio wishes to improve the reproducibility of the work that we publish. This form provides structure for consistency and transparency in reporting. For further information on Nature Portfolio policies, see our Editorial Policies and the Editorial Policy Checklist.

Statistics

For all statistical analyses, confirm that the following items are present in the figure legend, table legend, main text, or Methods section.

n/a	Confirmed	
☐	☒	The exact sample size (n) for each experimental group/condition, given as a discrete number and unit of measurement
☐	☒	A statement on whether measurements were taken from distinct samples or whether the same sample was measured repeatedly
☐	☒	The statistical test(s) used AND whether they are one- or two-sided<br>*Only common tests should be described solely by name; describe more complex techniques in the Methods section.*
☐	☒	A description of all covariates tested
☐	☒	A description of any assumptions or corrections, such as tests of normality and adjustment for multiple comparisons
☐	☒	A full description of the statistical parameters including central tendency (e.g. means) or other basic estimates (e.g. regression coefficient) AND variation (e.g. standard deviation) or associated estimates of uncertainty (e.g. confidence intervals)
☐	☒	For null hypothesis testing, the test statistic (e.g. F, t, r) with confidence intervals, effect sizes, degrees of freedom and P value noted<br>*Give P values as exact values whenever suitable.*
☒	☐	For Bayesian analysis, information on the choice of priors and Markov chain Monte Carlo settings
☒	☐	For hierarchical and complex designs, identification of the appropriate level for tests and full reporting of outcomes
☐	☒	Estimates of effect sizes (e.g. Cohen's d, Pearson's r), indicating how they were calculated

Our web collection on statistics for biologists contains articles on many of the points above.

Software and code

Policy information about availability of computer code

Data collection	There is no code or software used to collect the data in this study.
Data analysis	Bioinformatic analysis: FastQC (v0.11.8), Trimmomatic (v0.39), Shovill (v1.4.1), CheckM (v1.1.3), Prokka (v1.14.6), OrthoFinder (v2.5.1), MAFFT (v7.471), IQ-TREE (v1.6.12), PopCOGenT (released version: 2022), KofamScan (v1.3.0), Unicycler (v0.5.0), Flye (v2.6), Canu (v2.2), Pilon (v1.24), Prokka (v1.14.6), CheckM (v1.0.7), miComplete (v1.1.1), HMMER (v3.3), iTOL (v5), Papara (v2.5), IQ-Tree (v1.6.12), MAFFT (v7.471), IQ-TREE (v1.6.12), ecceTERA (v1.0.0), ANGST (v1.0.0), R (v3.5.1), DADA2 (v1.10.0).<br>Statistical analysis: R (v4.1.1). R packages: “vegan (v.2.7)”, “phytools (v2.5-1)”, “lme4 (v1.1-38)”, “emmeans (v2.0.1)”, “phytools (v2.5-2)”, “ape (v5.8-1)”, “optparse (v1.7.5)”.<br>See custom code along with analysis pipeline used in this study in the repository (https://github.com/444thLiao/Denitrification_Ruegeria).

For manuscripts utilizing custom algorithms or software that are central to the research but not yet described in published literature, software must be made available to editors and reviewers. We strongly encourage code deposition in a community repository (e.g. GitHub). See the Nature Portfolio guidelines for submitting code & software for further information.

Data

Policy information about availability of data

All manuscripts must include a data availability statement. This statement should provide the following information, where applicable:

- Accession codes, unique identifiers, or web links for publicly available datasets
- A description of any restrictions on data availability
- For clinical datasets or third party data, please ensure that the statement adheres to our policy

Raw sequencing data are available on NCBI under several BioProjects with the private access links for reviewers. The raw data for nirS amplicon and Ruegeria population-resolving ATP5B, parC, and nirS amplicons are available under NCBI BioProject ID: PRJNA1310737 (https://dataview.ncbi.nlm.nih.gov/object/PRJNA1310737?reviewer=5ms8d3b13dqlg4f11rmq9rnkj4), PRJNA1275610 (https://dataview.ncbi.nlm.nih.gov/object/PRJNA1275610?reviewer=nst2vifa08hqr8otcjvv39ge4l), and PRJNA1275576 (https://dataview.ncbi.nlm.nih.gov/object/PRJNA1275576?reviewer=g865emc830spbkko9b86qtcc35). Raw reads and assembly of 419 Ruegeria genomes (i.e., missing raw reads for 10 genomes) were available under the NCBI BioProject ID PRJNA1264799 (https://dataview.ncbi.nlm.nih.gov/object/PRJNA1264799?reviewer= mp7rulknapinfb95c9733nf72r). Raw reads and assembly of 34 Nanopore closed Ruegeria genomes were available under the NCBI BioProject ID PRJNA1275854 (https://dataview.ncbi.nlm.nih.gov/object/PRJNA1275854?reviewer=g4mffnb8b965tq6b2662cpt6i8). Additionally, 26 referenced Ruegeria genomes were deposited in the Zenodo (https://zenodo.org/records/18171395). Raw data from the 15N-stable isotope assay has been deposited in the public repository Zenodo (https://zenodo.org/records/18028409). All data will be publicly accessible upon publication.

Research involving human participants, their data, or biological material

Policy information about studies with human participants or human data. See also policy information about sex, gender (identity/presentation), and sexual orientation and race, ethnicity and racism.

Reporting on sex and gender	This research did not involve human participants.
Reporting on race, ethnicity, or other socially relevant groupings	This research did not involve human participants.
Population characteristics	This research did not involve human participants.
Recruitment	This research did not involve human participants.
Ethics oversight	This research did not involve human participants.

Note that full information on the approval of the study protocol must also be provided in the manuscript.

Field-specific reporting

Please select the one below that is the best fit for your research. If you are not sure, read the appropriate sections before making your selection.

☐ Life sciences ☐ Behavioural & social sciences ☒ Ecological, evolutionary & environmental sciences

For a reference copy of the document with all sections, see nature.com/documents/nr-reporting-summary-flat.pdf

Ecological, evolutionary & environmental sciences study design

All studies must disclose on these points even when the disclosure is negative.

Study description	This study identified and validated the presence of denitrifying “specialist” populations in Hong Kong hyper-eutrophicated coral reefs using combined population genomics, subspecies-resolution metabarcoding (resolving both the entire Ruegeria community and the denitrifying sub-community), and direct isotope-based activity assays.
Research sample	For Ruegeria culture-dependent study, we used five coral species Acropora solitaryensis, Acropora cf. samoensis, Platgyra acuta, Favites adbita, and Oulastrea crispata sampled from nine sites (Yam Tsai Wan (YTW), Sham Wan (SW), Lo Chau (LC), Bluff Island (BI), Ngo Mei Chau (NMC), Chek Chau (CC), Wong Wan Chau (WWC), Ninepine (NP), and Kiu Tsui Chau (KTC)) in Hong Kong SAR.<br>For Ruegeria culture-independent metabarcoding study, we used five coral species, i.e., Oulastrea crispata, Favites abdita, Leptastrea pruinosa, Porites lutea, and Platygyra acuta, collected along nine western to eastern Hong Kong reef sites, i.e., YTW, PC, SW, LC, NP, Sharp Island (SI), and Port Island (PI) in November 2022. These coral species were chosen due to their consistent prevalence along coastal Hong Kong waters.<br>For 15N-based denitrification activity measurement, we assessed four denitrifying specialist MCs (i.e., MC1, MC6, MC13, and MC18) and nine non-specialist MCs (i.e., MC0, MC3, MC7, MC12, MC17, MC19, MC20, MC21, and MC26) scattered and widely distributed in the genome tree.
Sampling strategy	For 15N-based denitrification activity measurement, we chose three isolates for each denitrifying specialist MC, one to three isolates for each denitrifying non-specialist MC based on isolate availability. Five biological replicates were used for each Ruegeria isolate. Sample size = 5 per isolates; Sample size = 12 for denitrifying specialists and 17 for non-specialists.

Data collection	Genome and metabarcoding sequencing were conducted at BGI, Hong Kong. Data were collected by Tianhua Liao (sequencing data analysis), Nan Xiang (sequencing data analysis and experiments on preparing sequencing samples), and Mei Xie (experiments on preparing sequencing samples).<br><br>15N-based N2O and N2 gas measurements were done by Richard Doucett from the stable isotope facility at UC-Davis, USA. Data were collected by Nan Xiang (gas experiment, isotopic data analysis), Chun Ho Mak (gas experiment), and Xiaowei Tang (gas experiment).
Timing and spatial scale	Genome data collection: time (year 2020 to 2022); frequency and periodicity (six times); spatial scale (nine sites including Yam Tsai Wan (YTW), Sham Wan (SW), Lo Chau (LC), Bluff Island (BI), Ngo Mei Chau (NMC), Chek Chau (CC), Wong Wan Chau (WWC), Ninepine (NP), and Kiu Tsui Chau (KTC)).<br>Metabarcoding data collection: time (2022 November); frequency and periodicity (once); spatial scale (seven sites including YTW, PC, SW, LC, NP, Sharp Island (SI), and Port Island (PI)).<br>15N-based stable isotope assay: time (2024 June to October and 2025 January to May); frequency and periodicity (twice). The first round of experiment is used to validate our protocol with two different dissolved oxygen levels. The second round is the experiment including all isolates shown in the Fig. 3 to exclude any potential experimental round-effect.
Data exclusions	Some of coral DNA samples failed in metabarcoding (ATP5B, parC, or nirS) amplifications, which were excluded from sequencing and subsequent analysis. They were not shown in the manuscript.
Reproducibility	We included three biological replicates for each coral sample. We included five biological replicates for each Ruegeria isolate samples under 15N-based stable isotope assay.
Randomization	For 15N-based stable isotope assay, all incubation chambers were randomly allocated to each replicate of the isolate. Chambers were randomly assigned to the positions in the shakers. To exclude the potential chamber effect, we fitted a linear mixed-effects model (LMM) defining the logarithm-transformed 46N2O and 30N2 values as the responsible variables, with MC identity and isolate ID as two fixed effects and biological replicate (chamber ID) as a random effect.
Blinding	During data acquisition and analysis of genomic sequencing part, blinding was not implemented. This study relied on high-throughput sequencing and standardized, automated bioinformatics/statistical workflows with pre-specified parameters and QC criteria, and the primary outcomes (e.g., genome assemblies, completeness/contamination metrics, gene annotation and orthogroup inference, phylogenetic inference, and amplicon ASV inference) were algorithmically generated rather than subjectively assessed. Group identities were used only for downstream comparative analyses and interpretation.<br>During data acquisition and analysis of 15N-based stable isotope part: the person generating measurements (e.g., running assays, sample collection, and data analysis) does not know the group label of each sample as we marked samples randomly by Arabic numerals.

Did the study involve field work? ☒ Yes ☐ No

Field work, collection and transport

Field conditions	Field works for coral sampling were conducted across three years with natural conditions varied widely. All coral samples were taken consistently from 3 meter in depth. The environmental conditions for sampling sites were detailed in Supplementary Table 1 as a reference.
Location	Coral reefs in Hong Kong SAR. Latitude: 22°08' N to 22°35' N, Longitude: 113°49' E to 114°31' E; In total of eleven western to eastern Hong Kong reef sites, i.e., Yam Tsai Wan (YTW), Sham Wan (SW), Lo Chau (LC), Bluff Island (BI), Ngo Mei Chau (NMC), Chek Chau (CC), Wong Wan Chau (WWC), Ninepine (NP), Kiu Tsui Chau (KTC), Sharp Island (SI), and Port Island (PI) were visited in this study .
Access & import/export	Coral samples were collected in compliance with local laws and sampling permissions.
Disturbance	We collected corals with a size of 5 to 10 cm2 for each fragment to minimize the disturbances to natural coral reefs.

Reporting for specific materials, systems and methods

We require information from authors about some types of materials, experimental systems and methods used in many studies. Here, indicate whether each material, system or method listed is relevant to your study. If you are not sure if a list item applies to your research, read the appropriate section before selecting a response.

Materials & experimental systems

n/a	Involved in the study
☒	☐ Antibodies
☒	☐ Eukaryotic cell lines
☒	☐ Palaeontology and archaeology
☐	☒ Animals and other organisms
☒	☐ Clinical data
☒	☐ Dual use research of concern
☒	☐ Plants

Methods

n/a	Involved in the study
☒	☐ ChIP-seq
☒	☐ Flow cytometry
☒	☐ MRI-based neuroimaging

Animals and other research organisms

Policy information about studies involving animals; ARRIVE guidelines recommended for reporting animal research, and Sex and Gender in Research

Laboratory animals	This study did not involve any laboratory animals.
Wild animals	In total of eight coral species (Acropora solitaryensis, Acropora cf. samoensis, Platgyra acuta, Favites adbita, Oulastrea crispata, Leptastrea pruinosa, Porites lutea, and Platygyra acuta) were sampled in this study. Wild coral animals were collected with a chisel and hammer, placed in sterilized bags on ice, and immediately transported to the laboratory. Remaining coral samples after experimental usage were all stored in -80 oC freezer.
Reporting on sex	Sex is not considered as a factor in our experimental design.
Field-collected samples	Coral samples were processed directly after sampling.
Ethics oversight	No ethical approval or guidance was required in this study, as the protocols used in this study all follow previous published high-profile papers.

Note that full information on the approval of the study protocol must also be provided in the manuscript.

Plants

Seed stocks	No plant sample was used in this study.
Novel plant genotypes	No plant sample was used in this study.
Authentication	No plant sample was used in this study.
